# Could we detect intraventricular hemorrhage before it happens?

**DOI:** 10.1038/s41390-024-03202-7

**Published:** 2024-04-09

**Authors:** Kelly Q. Zhou, Simerdeep K. Dhillon, Joanne O. Davidson, Alistair J. Gunn

**Affiliations:** Department of Physiology, The University of Auckland, Auckland, New Zealand

Germinal matrix intraventricular hemorrhage (IVH) is a serious complication of extremely preterm birth.^1^ Its effects can be devastating, with high risks of death and severe neurodevelopmental disability, mainly after higher grade of hemorrhage.^2,3^ IVH most often occurs in the early hours or days after birth.^2,4^ Infants with IVH can be detected non-invasively on serial cranial ultrasounds, but by definition after it is present.^5^ A predictive biomarker is vital to help develop preventative strategies for IVH.

There is growing evidence that useful biological signals can be detected. For example, a small single-center prospective study of urine samples collected from preterm infants with IVH (*n* = 7) and without IVH (*n* = 11), analyzed with targeted liquid chromatography-tandem mass spectrometry,^6^ showed that 20 of the 40 metabolites assessed were significantly different between the groups. The altered profile suggested a shift toward anaerobic metabolism and mitochondrial dysfunction, likely associated with hypoxia-ischemia and hypoperfusion. However, infants in the IVH group had lower gestational age that the controls, leaving the possibility that some of the changes may have been due to immaturity.

In this issue, Ducatez et al. conducted a study of high-throughput assessment of cord blood in a prospective case-control study of 26 patients with germinal matrix-IVH and 60 control premature newborns at Rouen University Hospital from 2015 to 2020. This study reported that IVH was associated with upregulation of leukocyte-associated immunoglobulin-like receptor 2 (LAIR-2), tumor necrosis factor-related apoptosis-inducing ligand receptor 2 (TRAIL-R2), placental growth factor (PGF), and Brother of CDO (BOC) cell adhesion associated protein.^7^ There was also a decrease of medium-chain long acylcarnitine C10:1 and 21 different phosphatidylcholines in infants with IVH. The reader should consider some limitations of these findings, particularly that it is vital to replicate the underlying observation. The study had a small sample size, including only 26 infants with variable IVH severities. Further, there was potential confounding, in that a higher proportion of babies with IVH required invasive positive pressure ventilation compared with controls (48% vs. 5.3%), and after adjusting for this, there was no significant association.

This study excluded infants with IVH on early cranial ultrasound, supporting that these biomolecular changes might be useful prognostic markers for the risk of a particular infant to develop IVH. Further analysis of early and late onset IVH and sampling at different times is now needed to provide insight into how these proteins and metabolites change over time with IVH. Well powered studies will be needed to dissect changes in cord blood biomolecules between IVH and underlying conditions, such as fetal growth restriction.^8^ Preclinical and clinical studies are needed to assess if the IVH associated proteomic and metabolic changes are a consequence or causal, and to evaluate the relevance of these biomolecules in pathogenesis. Additionally, Ducatez et al. still used a targeted approach, and so it is possible that other novel biomolecules and markers may have been missed.

To further validate potential biomarkers it will be critical to assess them in conjunction with clinical physiology. For example, changes in mean arterial pressure and cerebral blood flow are the most important physiological parameters involved in the pathogenesis of IVH. While there were no significant differences in clinical characteristics between the study groups, the association between cord blood biomarkers and early postnatal cardiovascular parameters was not assessed.

Although omics are not feasible at an individual patient level, once potential biomarkers have been validated by multiple studies, it would support the development of bedside assays. These would be useful for rapid identification of babies who are at risk for developing IVH. Importantly for the future, there are currently no available treatment options for infants with IVH. Current animal models to study the pathophysiology and treatment of IVH involve rabbits, mice and rats, each of which have limitations as reviewed.^9^ These models involve injection of glycerol (intraperitoneal), blood or blood derivatives (intraventricular), and collagenase (intraventricular). Although these approaches result in brain atrophy, white matter impairments, inflammation and motor and cognitive impairments,^9^ they do not directly recapitulate the pathophysiological events that lead to IVH. In particular, there is evidence that autoregulation is not fully developed in preterm infants, so that they cannot adequately compensate for fluctuations in cerebral blood flow, thus making them more vulnerable to IVH.^10^ More physiological, large animals models of IVH are needed, where germinal matrix vessels would be directly injured by hypoxia-ischemia or hyperperfusion.

In summary, the findings of Ducatez et al. and others are promising, but direct replication in well powered studies to confirm that the proposed biomarkers of risk of IVH are reliable is now needed. Unbiased omics studies are likely to be a valuable tool to confirm such biomarkers, and in turn to support targeted recruitment of babies for randomized control trials.

## References

[1] Leijser, L. M. & de Vries, L. S. Preterm brain injury: germinal matrix-intraventricular hemorrhage and post-hemorrhagic ventricular dilatation. *Handb. Clin. Neurol.* **162**, 173–199 (2019). 31324310 10.1016/B978-0-444-64029-1.00008-4

[2] Ballabh, P. Pathogenesis and prevention of intraventricular hemorrhage. *Clin. Perinatol.* **41**, 47–67 (2014). 24524446 10.1016/j.clp.2013.09.007 PMC3925310

[3] Gilard, V. et al. Post hemorrhagic hydrocephalus and neurodevelopmental outcomes in a context of neonatal intraventricular hemorrhage: an institutional experience in 122 preterm children. *BMC Pediatr.* **18**, 288 (2018). 30170570 10.1186/s12887-018-1249-x PMC6119335

[4] Papile, L. A., Burstein, J., Burstein, R. & Koffler, H. Incidence and evolution of subependymal and intraventricular hemorrhage: a study of infants with birth weights less than 1,500 gm. *J. Pediatr.* **92**, 529–534 (1978). 305471 10.1016/s0022-3476(78)80282-0

[5] Parodi, A., Govaert, P., Horsch, S., Bravo, M. C. & Ramenghi, L. A. Cranial ultrasound findings in preterm germinal matrix haemorrhage, sequelae and outcome. *Pediatr. Res.* **87**, 13–24 (2020). 32218535 10.1038/s41390-020-0780-2 PMC7098890

[6] Sarafidis, K. et al. Targeted urine metabolomics in preterm neonates with intraventricular hemorrhage. *J. Chromatogr. B* **1104**, 240–248 (2019). 10.1016/j.jchromb.2018.11.024 30530117

[7] Ducatez, F. et al. New insights and potential biomarkers for intraventricular hemorrhage in extremely premature infant, case-control study. *Pediatr. Res.*10.1038/s41390-024-03111-9 (2024). 10.1038/s41390-024-03111-9 38467704

[8] Chao de la Barca, J. M. et al. A metabolomic profiling of intra-uterine growth restriction in placenta and cord blood points to an impairment of lipid and energetic metabolism. *Biomedicines* **10**, 1411 (2022). 35740432 10.3390/biomedicines10061411 PMC9220006

[9] Atienza-Navarro, I., Alves-Martinez, P., Lubian-Lopez, S. & Garcia-Alloza, M. Germinal matrix-intraventricular hemorrhage of the preterm newborn and preclinical models: inflammatory considerations. *Int. J. Mol. Sci.* **21**, 8343 (2020). 33172205 10.3390/ijms21218343 PMC7664434

[10] Lou, H. C., Lassen, N. A. & Friis-Hansen, B. Impaired autoregulation of cerebral blood flow in the distressed newborn infant. *J. Pediatr.* **94**, 118–121 (1979). 758388 10.1016/s0022-3476(79)80373-x

